# Macrophage migration inhibitory factor (MIF) inhibitor 4-IPP downregulates stemness phenotype and mesenchymal trans-differentiation after irradiation in glioblastoma multiforme

**DOI:** 10.1371/journal.pone.0257375

**Published:** 2021-09-13

**Authors:** Shin Heon Lee, Hyung Joon Kwon, Saewhan Park, Chan Il Kim, Haseo Ryu, Sung Soo Kim, Jong Bae Park, Jeong Taik Kwon

**Affiliations:** 1 Department of Medicine, Graduate School, Chung-Ang University, Seoul, Korea; 2 Department of Neurosurgery, Samsung Medical Center, Sungkyunkwan University School of Medicine, Seoul, Korea; 3 Department of Cancer Control and Population Health, Graduate School of Cancer Science and Policy, National Cancer Center, Goyang, Korea; 4 Department of Cancer Biomedical Science, Graduate School of Cancer Science and Policy, National Cancer Center, Goyang, Korea; 5 Department of Clinical Research, Research Institute and Hospital, National Cancer Center, Goyang, Korea; 6 Graduate School of Cancer Science and Policy R&D Foundation, National Cancer Center, Goyang, Korea; 7 Department of Neurosurgery, Chung-Ang University Hospital, Chung-Ang University College of Medicine, Seoul, Korea; Bauer Research Foundation, UNITED STATES

## Abstract

Radiation therapy is among the most essential treatment methods for glioblastoma multiforme (GBM). Radio-resistance and cancer stem cell properties can cause therapeutic resistance, cancer heterogeneity, and poor prognoses in association with GBM. Furthermore, the GBM subtype transition from proneural to the most malignant mesenchymal subtype after radiation therapy also accounts for high resistance to conventional treatments. Here, we demonstrate that the inhibition of macrophage migration inhibitory factor (MIF) and D-dopachrome tautomerase (DDT) by 4-iodo-6-phenylpyrimidine (4-IPP), a dual inhibitor targeting MIF and DDT, downregulates stemness phenotype, intracellular signaling cascades, mesenchymal trans-differentiation, and induces apoptosis in proneural glioma stem cells (GSCs). In an analysis of The Cancer Genome Atlas, high MIF and DDT expression were associated with poor prognosis. GSC growth was effectively inhibited by 4-IPP in a time- and dose-dependent manner, and 4-IPP combined with radiation therapy led to significantly reduced proliferation compared with radiation therapy alone. The expression of stemness factors, such as Olig2 and SOX2, and the expression of pAKT, indicating PI3K signaling pathway activation, were decreased in association with both 4-IPP monotherapy and combination treatment. The expression of mesenchymal markers, TGM2 and NF-κB, and expression of pERK (indicating MAPK signaling pathway activation) increased in association with radiation therapy alone but not with 4-IPP monotherapy and combination therapy. In addition, the combination of 4-IPP and radiation therapy significantly induced apoptosis compared to the monotherapy of 4-IPP or radiation. In vivo results demonstrated a significant tumor-suppressing effect of 4-IPP when combined with radiation therapy. Collectively, our results showed that the targeted inhibition of MIF and DDT has the potential to strengthen current clinical strategies by enhancing the anticancer effects of radiation therapy.

## Introduction

Glioblastoma multiforme (GBM) is the most malignant type of brain tumor, with rapid recurrence and an extremely low survival rate [1]. For GBM, radiation therapy is among the most essential treatment methods, along with chemotherapy [2]. However, recurrence occurs frequently after radiation therapy due to the survival of radio-resistant tumor cells, inflammatory responses, immune suppression, and neo-vascularization of the irradiated areas [3,4].

Macrophage migration inhibitory factor (MIF) exerts multimodal functions in glioma, including proliferation, migration, angiogenesis promotion, inhibition of apoptosis, and immune evasion properties [5]. MIF is triggered by autocrine and paracrine signals by the tumor, and its expression level increases with the higher-grade tumors [6]. Evaluation of bioinformatics databases has shown significantly higher levels of MIF in GBM compared with non-tumor tissues, and MIF expression has been associated with poor prognoses and early recurrence in association with gliomas [7,8].

Cancer stem cell (CSC) property can cause therapeutic resistance to, and heterogeneity of, GBM in the context of developing therapeutics [9]. MIF plays a role in the stemness properties of gliomas [10]. In two previous studies, brain tumor–initiating cells (BTICs) and glioma stem cells (GSCs) were shown to express higher levels of MIF compared with non–stem cells, and a correlation between MIF and stemness was demonstrated [11,12]. Also, secreted MIF from CSCs activates and protects myeloid-derived suppressor cells and inhibits immunological rejection [13]. Additionally, MIF directly inhibits p53, maintaining the tumorigenic capacity of BTICs [11]. Furthermore, MIF is secreted by ionizing radiation and other DNA-damaging stresses in cancer cells [5]. Thus, MIF can induce immune escape and weaken the beneficial effects of treatment.

DNA methylation, inflammatory microenvironments, reactive oxygen species, and signal transducer and activator of transcription 3 (STAT3) activation are known to contribute to the mesenchymal trans-differentiation of GBM [14]. The transition of GBM from the proneural subtype to the mesenchymal subtype after radiation therapy is closely associated with gaining resistance to conventional treatments and poor prognosis [15]. Mesenchymal trans-differentiation is driven by the activation of a limited set of master regulators; nuclear factor kappa-light-chain-enhancer of activated B cells (NF-κB) is the upstream regulator of these master regulators and is closely associated with mesenchymal trans-differentiation [16]. MIF binds to the NF-κB activation inhibitor thioredoxin interacting protein (TXNIP) to induce the activation of NF-κB [17]. Therefore, MIF inhibition may reduce resistance to radiation therapy by downregulating stem cell properties and NF-κB-mediated mesenchymal trans-differentiation after irradiation.

The MIF signaling cascade begins with the binding of MIF to the extracellular domain of the non-polymorphic type II integrated membrane protein CD74 [18]. MIF-induced signaling acts through a few downstream pathways, including the mitogen-activated protein kinase (MAPK) and phosphoinositide 3-kinase (PI3K) signaling pathways, which play roles in oncogenesis [18]. D-dopachrome tautomerase (DDT) participates in the MIF cascade by acting as a second ligand for CD74, which exhibits a strong structural similarity to, and a close gene distance with, MIF [19]. Functionally, DDT and MIF overlap significantly in terms of cell survival control, tumor formation, and tumor migration, while DDT and MIF additively regulate the growth and survival of cancer cells [19]. The double inhibition of DDT and MIF also reflects a synergistic effect, since DDT can compensate for MIF single inhibition [20]. Therefore, efforts to inhibit MIF signaling in cancer should target DDT together. The dual inhibitor 4-iodo-6-phenylpyrimidine (4-IPP) is capable of targeting MIF and DDT together and forms a covalent bond with Pro-1 of both proteins to alter the structure and prevent function [21]. Thus, it is possible to inhibit cancer cell growth by targeting DDT and MIF with a single molecule, which may provide more efficient therapeutic benefits to patients with glioma [21].

In this study, we demonstrated that double inhibition of MIF and DDT suppresses stemness phenotype, intracellular signal transduction cascades, mesenchymal trans-differentiation, and induces apoptosis in GSCs, potentially contributing to enhanced radio-sensitivity. We investigated the effect of 4-IPP on proneural GSCs.

## Materials and methods

### Cohort analysis

Patient survival data and MIF and DDT gene expression levels were obtained using The Cancer Genome Atlas (TCGA) Lower Grade Glioma and Glioblastoma (GBMLGG) cohort dataset (n = 694) from the University of California Santa Cruz (UCSC) Xena platform (https://xenabrowser.net/datapages/) [8]. Kaplan-Meier plots were generated to analyze overall survival for 175 patients with low gene expression (quartile 1) and 175 patients with high gene expression (quartile 4) for MIF and DDT, respectively. We also analyzed overall survival in 96 patients with low gene expression for both MIF and DDT (quartile 1) and 116 patients with high gene expression for both MIF and DDT (quartile 4). Pearson correlation coefficient was carried out to explore the correlation between MIF and DDT expression.

### Cell culture and reagents

Patient-derived GSCs (528NS and 448T) were maintained in Dulbecco’s modified Eagle’s medium (DMEM)/F-12 supplemented with B27 (Invitrogen), epidermal growth factor (EGF; R&D Systems), and basic fibroblast growth factor (bFGF; R&D Systems). GSC 528NS was obtained from Dr. Ichiro Nakano (University of Alabama at Birmingham, Birmingham, AL, USA) and GSC 448T was obtained from Dr. Do-Hyun Nam (Samsung Medical Center, Seoul, Korea). The cell lines were used within 10–30 passages of their establishment from primary cells. We purchased 4-IPP from Tocris Biosciences. All cells were maintained in culture for <6 months after receipt and repeatedly screened for mycoplasma with the Universal Mycoplasma Detection Kit (ATCC) according to the manufacturer’s protocol.

### Ionizing radiation

The cells were irradiated with a single fraction X-ray source with a dose of 6 Gy at room temperature using the XenX irradiator (Xstrahl Ltd., Camberley, UK). Ninety minutes after irradiation, the cells were treated with 50 μM 4-IPP. After 48 hours, the cells were collected to calculate the effects of different treatments.

### Cell proliferation assay

GSCs were plated at 10^3^ cells/well densities in 96-well plates containing DMEM/F-12 supplemented with B27, EGF, and bFGF for in vitro 3-(4,5-dimethylthiazol-2-yl)-2,5-diphenyl tetrazolium bromide (MTT) proliferation assays. The luminescence of viable cells was detected using the CellTiter-Glo Luminescent Cell Viability Assay Kit (Promega) according to the manufacturer’s instructions. The CellTiter-Glo luminescent cell viability assay is a homogenous method of determining the number of viable cells in culture based on the quantitation of the ATP present, which signals the presence of metabolically active cells. The luminescence signal was detected using a SpectraMax L Microplate Reader (Molecular Devices Inc., Sunnyvale, CA, USA) according to the manufacturer’s instructions.

Chemical interactions between MTT and 4-IPP were examined to evaluate the possible interference in absorbance value. According to the method by Ulukaya et al., the optical density value change between the drug-containing medium and the drug-free medium was evaluated, and the result was defined as the percent of variation using the following formula: variation (%) = (absorbance of drug-containing medium–absorbance of blank)/absorbance of blank * 100 (absorbance at 470 nm) [22]. Referring to the previous study, increases or decreases larger than 10% were considered significant [22].

### Immunoblot analysis

Proteins were extracted with radioimmunoprecipitation assay buffer with complete protease inhibitors (Roche), separated by electrophoresis, transferred to polyvinylidene fluoride membranes (Millipore), and blocked with 5% skim milk (BD Biosciences). Primary antibodies against MIF (Abcam), serine 473-phosphorylated protein kinase B (pAKT; Cell Signaling), phosphorylated extracellular signal-regulated kinase (pERK; Cell Signaling), oligodendrocyte transcription factor 2 (Olig2; Abcam), sex-determining region Y-box 2 (SOX2; R&D Systems), transglutaminase 2 (TGM2; Millipore), NF-κB (Santa Cruz), CCAAT/enhancer-binding protein beta (C/EBP-β; Santa Cruz), vinculin (Sigma-Aldrich), and beta-actin (β-actin; Santa Cruz) were incubated overnight at 4°C Immunoreactive bands were visualized using peroxidase-labeled affinity-purified secondary antibodies (KPL) and the Amersham ECL Prime Western blotting detection reagent (GE Healthcare). The concentration of the primary antibody was set as 1:1000 and that of the secondary antibody was set as 1:5000-fold dilution throughout all experiments.

### In vivo study

A total of 16 BALB/c nude mice were purchased from Orient Bio Inc. (Seongnam, Korea) and used to generate xenograft tumors. Only female mice were included in the experiment because male mice incline toward dominant behavior and are more difficult to maintain. The sample size was calculated using Mead’s resource equation method. The mice were 6 weeks old, and they weighed between 18 and 20 g when they were subcutaneously injected with tumor cells. Cells were transplanted following resuspension in DMEM/F-12 with B27, EGF, and bFGF for the subcutaneous xenograft mouse model.

Once the mice were anesthetized with Zoletil 50 (Virbac), their flanks were injected with 2 × 10^6^ 528NS cells in 100 μL of medium and equivalent amounts of Matrigel (Corning). In preparation for the possibility of tumor formation failure, the flank injections were performed bilaterally. In six subjects, xenograft tumors formed in one flank. One subject with outlying tumor growth was excluded from group allocation. Three weeks after injection, the mice were randomly assigned to four treatment groups: control (n = 3), 4-IPP (n = 4), radiation (n = 4), and combination (n = 4) so that there six tumor masses were included in each group and so that the mean tumor volume would be approximately 80 mm^3^ in each group. Tumor size was measured twice per week using electronic calipers to measure two diameters. Tumor size was defined by the following formula: length × width^2^ × 0.5. All measurements were conducted in blinded conditions and repeated at least three times.

Before receiving any intervention, all mice were acclimatized for at least 2 weeks in our animal facility. For the appropriate environment, housing, and management of laboratory mice, temperature and humidity were set to 22±1°C and 50±20%, respectively, and were recorded every 10 minutes. The operation was monitored 24 hours per day by a central monitoring system. All mice were provided with food (Altromin GmbH, Lage, Germany) and autoclaved reverse osmosis water while being kept in an individually ventilated cage rack (Three-Shine Inc., Daejeon, Korea) with Nestlets (Ancare, Bellmore, NY, USA) provided for environmental enrichment. Food and water intake were monitored daily to determine the health of the mice, and mice found to be unhealthy through observations of abnormal posture, decreased activity, or weight loss were immediately planned to be euthanized. Additionally, any mouse with a single tumor size measurement of at least 2000 mm^3^ was designated for euthanization.

The mean tumor volume at the start of the drug administration was approximately 250 mm^3^. Daily 4-IPP administration (5 mg/kg) proceeded via intraperitoneal injection. Control mice received vehicle (dimethyl sulfoxide) treatment. Radiation treatment was administered at a dose of 2.5 Gy for 4 days using the XenX irradiator when the mean tumor volume reached approximately 500 mm^3^. Mice were monitored daily, and there were no signs of severe illness and no deaths caused by experimental procedures throughout this study. When the single tumor mass of the fastest-growing mouse reached over 2000 mm^3^ (37 days after the cell injections), all mice were sacrificed by spinal dislocation after CO_2_ gas anesthesia following our institutional guidelines. Tumors were extracted, collected for each experimental group, and analyzed for tumor size and weight.

### Histology and immunohistochemical staining

To facilitate the observation of the histologic features, extracted tumors were fixed with 4% paraformaldehyde for 24 h at 4°C, sectioned at a thickness of 4 μm using an essential microtome (Leica RM2125 RTS, Leica Biosystems, Nussloch, Germany), and stained with hematoxylin (Dako) and 0.25% eosin (Merck). Before immunohistochemical (IHC) staining, tissue sections were subjected to antigen retrieval processes using citrate buffer (pH 6.0), and endogenous peroxidase was blocked via incubation with 3% hydrogen peroxide. Tissue sections were then incubated overnight at 4°C in a humidified chamber with the primary antibody for Olig2 (1:500), SOX2 (1:500), EGFR (1:500), pERK (1:500), pAKT (1:500), TGM2 (1:500), NF-κB (1:500), cleaved caspase-3 (1:500; Cell Signaling), and Ki-67 (1:3000; Abcam), and diluted with antibody diluent buffer (IHC World). The incubation of the corresponding secondary antibody was performed using the Polink-1 polymeric horseradish peroxidase (HRP) Detection Kit (GBI Labs) according to the manufacturer’s instructions. To avoid nonspecific binding in mouse tissue, we used Mouse Elite Peroxidase kits (Vector Laboratories). Tissue sections for 3,3′-diaminobenzidine (DAB) staining were developed using DAB (Vector Laboratories) as the chromogen.

For the evaluation of the degree of apoptosis and proliferation, five separate areas of cleaved caspase-3- and Ki-67-stained tissues were randomly selected with magnification using the Aperio ImageScope v.12.4.3 software (Leica Biosystems, Nussloch, Germany). High-necrotic regions were excluded for analysis. Cells positive for cleaved caspase-3 were counted and the percentage of Ki-67-positive stained area was calculated per selected area. The results were quantified by Image J software (National Institutes of Health, Bethesda, MD, USA) and assessed by two independent observers who were blinded to the treatment. The mean values of selected areas were calculated and used to determine the degree of apoptosis and proliferation.

### Ethical considerations

This study was reviewed and approved by the Institutional Animal Care and Use Committee of the National Cancer Center Research Institute (NCCRI) (Approval number: NCC-17-402). The NCCRI is an Association for Assessment and Accreditation of Laboratory Animal Care International–accredited facility and abides by the Institute of Laboratory Animal Resources guidelines. All surgical procedure was performed under anesthesia using Zoletil 50, and all efforts were made to minimize suffering.

### Statistical analysis

GraphPad Prism, version 8 (GraphPad Software Inc., San Diego, CA, USA) and Microsoft Excel 2019 (Microsoft Corp., Redmond, WA, USA) were used for all statistical analyses. Statistical comparisons were performed using Student’s t-test or two-way analysis of variance followed by Holm-Sidak testing. Quartiles Kaplan-Meier survival analysis was used to estimate the survival distributions. The log-rank test was used to assess for statistical significance in comparisons between the stratified survival groups. Pearson correlation coefficient was used to evaluate the correlation between MIF and DDT expression. P values less than 0.05 were considered statistically significant.

## Results

### MIF and DDT overexpression is associated with decreased survival in glioma patients

**Fig 1:**
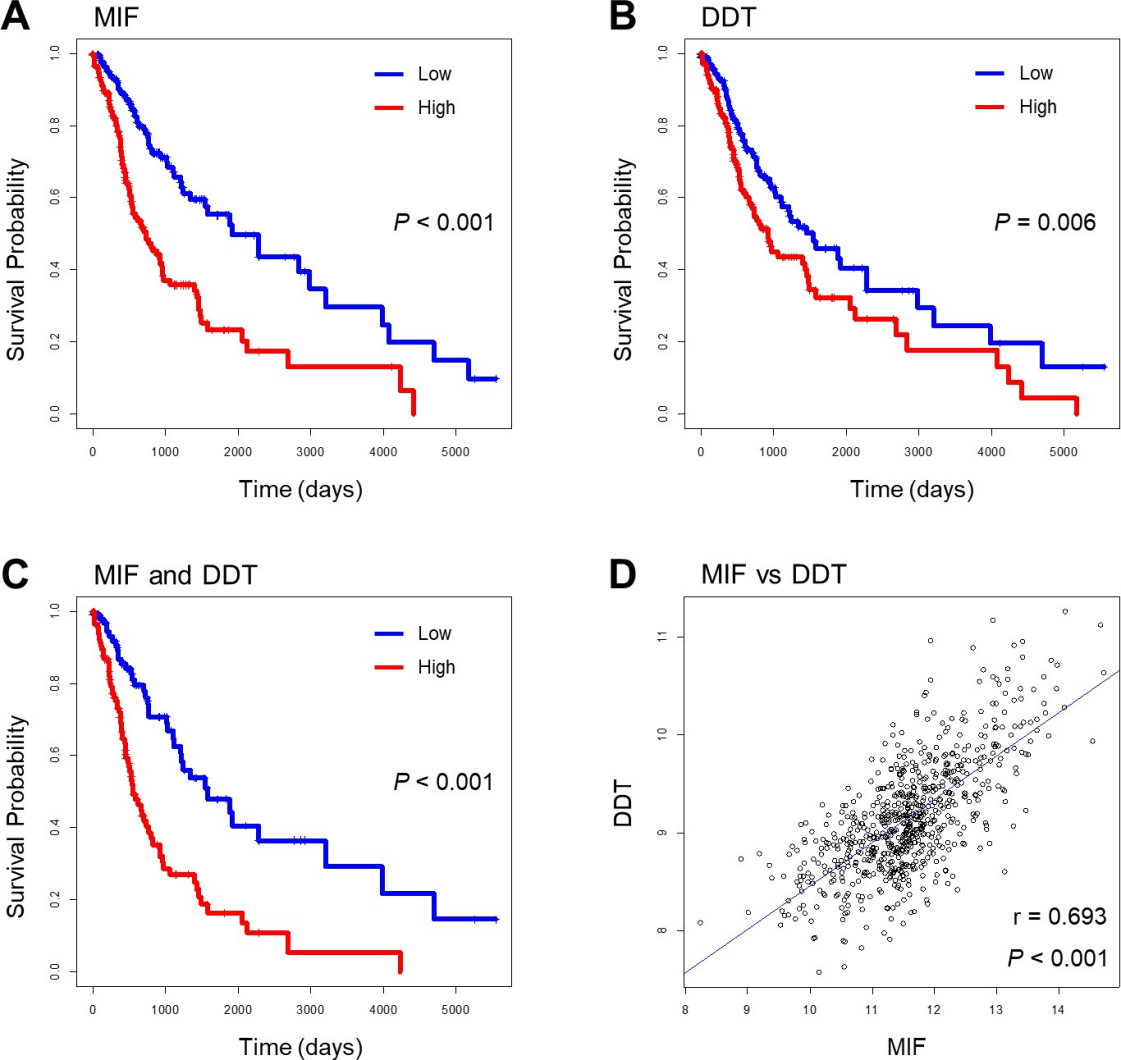
Kaplan-Meier survival curve and Pearson correlation coefficient based on the categorization of MIF and DDT expressions in the UCSC Xena platform.

To analyze the clinical importance of MIF and DDT expression in gliomas, we first investigated the relationship of MIF and DDT expression with the survival of glioma patients using TCGA-GBMLGG cohort dataset (n = 694) from the UCSC Xena platform. The quartiles Kaplan-Meier survival plots showed that the MIF and DDT RNA levels were negatively associated with patient survival (Fig 1A–1C). The glioma groups with high MIF and DDT expression levels, respectively, had a poorer prognosis than the glioma groups with low expression levels (Fig 1A and 1B). In addition, the glioma group with both high MIF and DDT expression also had a poorer prognosis than the glioma group with low expression of both MIF and DDT (Fig 1C). Pearson correlation coefficient indicated that MIF expression level was positively correlated with DDT expression level (Fig 1D).

### 4-IPP inhibits cell growth and enhances the therapeutic effects of radiation in GSCs

**Fig 2:**
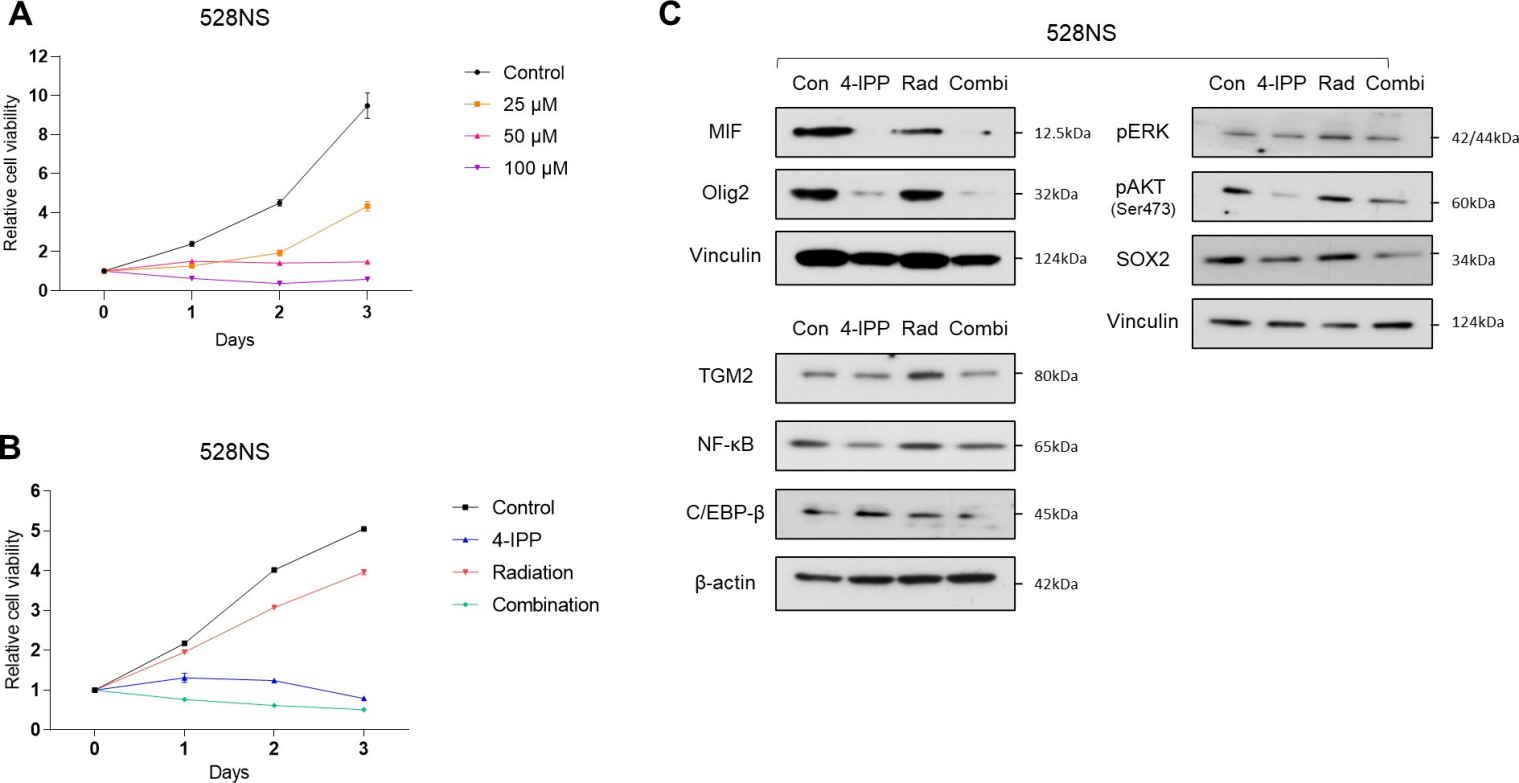
GSC after various treatments. (A) In vitro cytotoxicity test of 4-IPP in a time- and dose-dependent manner. (B) Survival curves of 528NS cells with various treatments are indicated. The effects of treatment were evaluated after treatment with the single and combinational application of 50 μM 4-IPP and X-ray irradiation (6Gy). (C) Protein expression by Western blot in 528NS cells when treated by single or combinational administration of 4-IPP and radiation.

Administration of 4-IPP did not cause any significant interference in the absorbance of the MTT assay. The mean variation in absorbance value was within the acceptable range (S1A and S1B Fig). In vitro cytotoxicity of 4-IPP was evaluated in GSCs via MTT assay. The 528NS cells showed effective growth inhibition by 4-IPP (Fig 2A). Growth curves of 448T cells are shown in S2 Fig. GSC growth was effectively inhibited by 4-IPP in a time- and dose-dependent manner. The factors representing stemness including Olig2 and SOX2 were evaluated via Western blot analysis, and the expressions were reduced in two kinds of GSCs by 4-IPP in a dose-dependent manner (S3A and S3B Fig).

The effect of 4-IPP on single and combinational applications with radiation therapy was evaluated in 528NS cells. Radiation plus 4-IPP led to significantly stronger inhibition of proliferation when compared with radiation alone in 528NS cells (Fig 2B). To address the functional significance of MIF and DDT inhibition, Western blot analysis was applied to evaluate the possible mechanism of 4-IPP in 528NS cells. Fig 2C shows the Western blot results of 528NS cells treated with different regimens. The expression of stemness factors, such as Olig2 and SOX2, was maintained in the radiation-alone group, and the expression of pAKT, a protein acting on the PI3K signaling pathway, was also maintained in the radiation-alone group. However, the expression of all of these factors decreased in the 4-IPP treatment and combination treatment groups. The expression of TGM2 and NF-κB, indicating mesenchymal trans-differentiation, and the expression of pERK from the MAPK signaling pathway increased in the radiation-alone group but did not increase in the 4-IPP and combination treatment groups. The expression of C/EBP-β, another protein indicating mesenchymal trans-differentiation did not significantly change in the 4-IPP or radiation-alone groups but decreased in the combination group (Fig 2C).

### 4-IPP suppresses stemness phenotype and downregulates mesenchymal trans-differentiation

**Fig 3:**
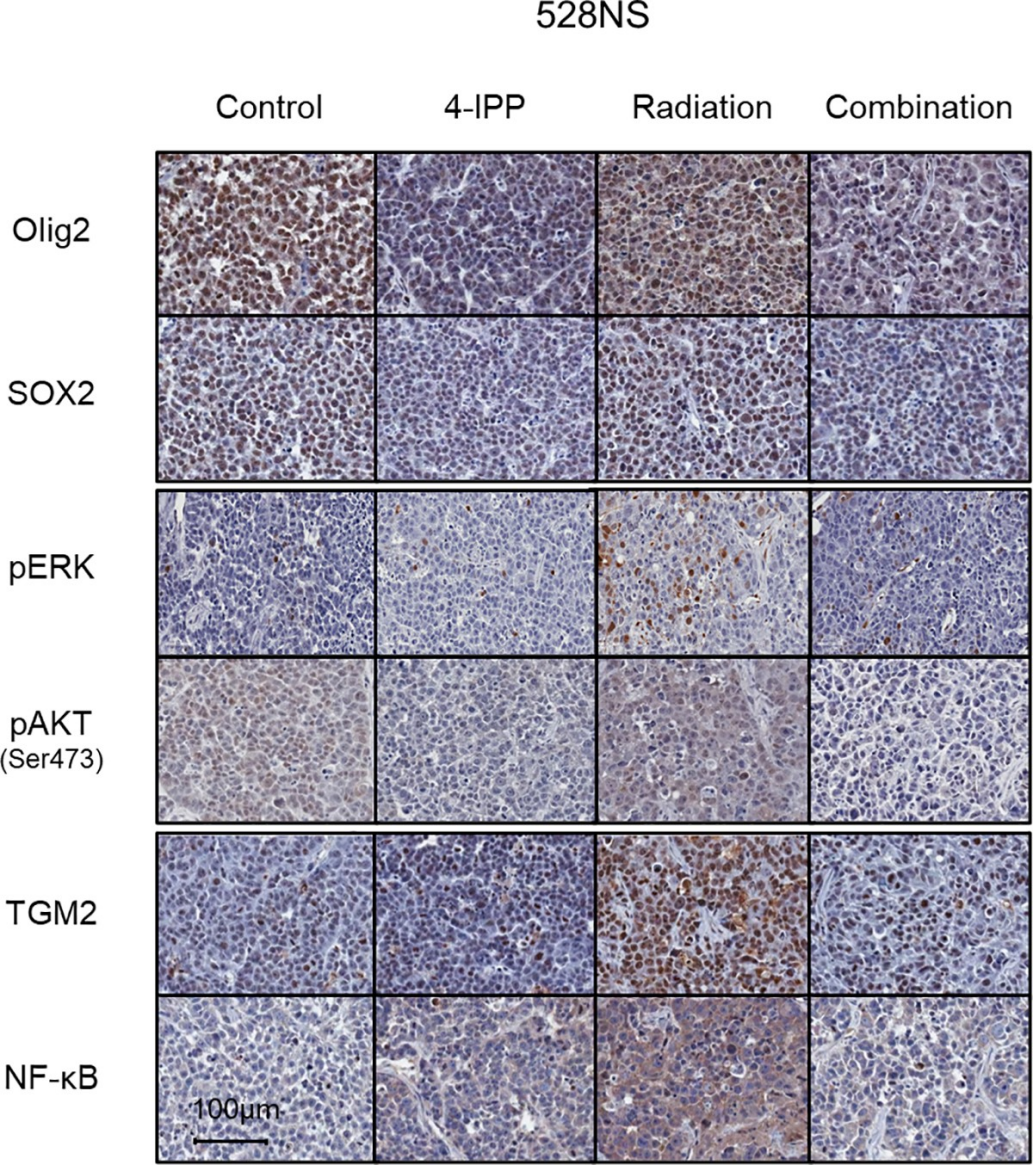
Immunohistochemical analysis of subcutaneous xenograft mouse model. Scale bar, 100 μm.

The effect of single and combinational applications of 4-IPP and radiation therapy on stemness and mesenchymal trans-differentiation was evaluated via IHC analysis of a xenograft mouse model established using 528NS cells. IHC analysis demonstrated that Olig2, SOX2, and pAKT were maintained after irradiation, and pERK, TGM2, and NF-κB increased in the radiation-only treatment group (Fig 3). The expression of Olig2, SOX2, and pAKT decreased with 4-IPP alone and 4-IPP plus radiotherapy. In addition, the expression of pERK, TGM2, and NF-κB decreased in the combination treatment in contrast to an increase in radiation-alone treatment (Fig 3). Similar to the Western blot results, factor expression indicating stemness, MAPK and PI3K cascade activation, and mesenchymal trans-differentiation after radiation was suppressed in association with 4-IPP administration (Fig 3).

### Influence of 4-IPP in apoptosis and proliferation rate of GSCs

**Fig 4:**
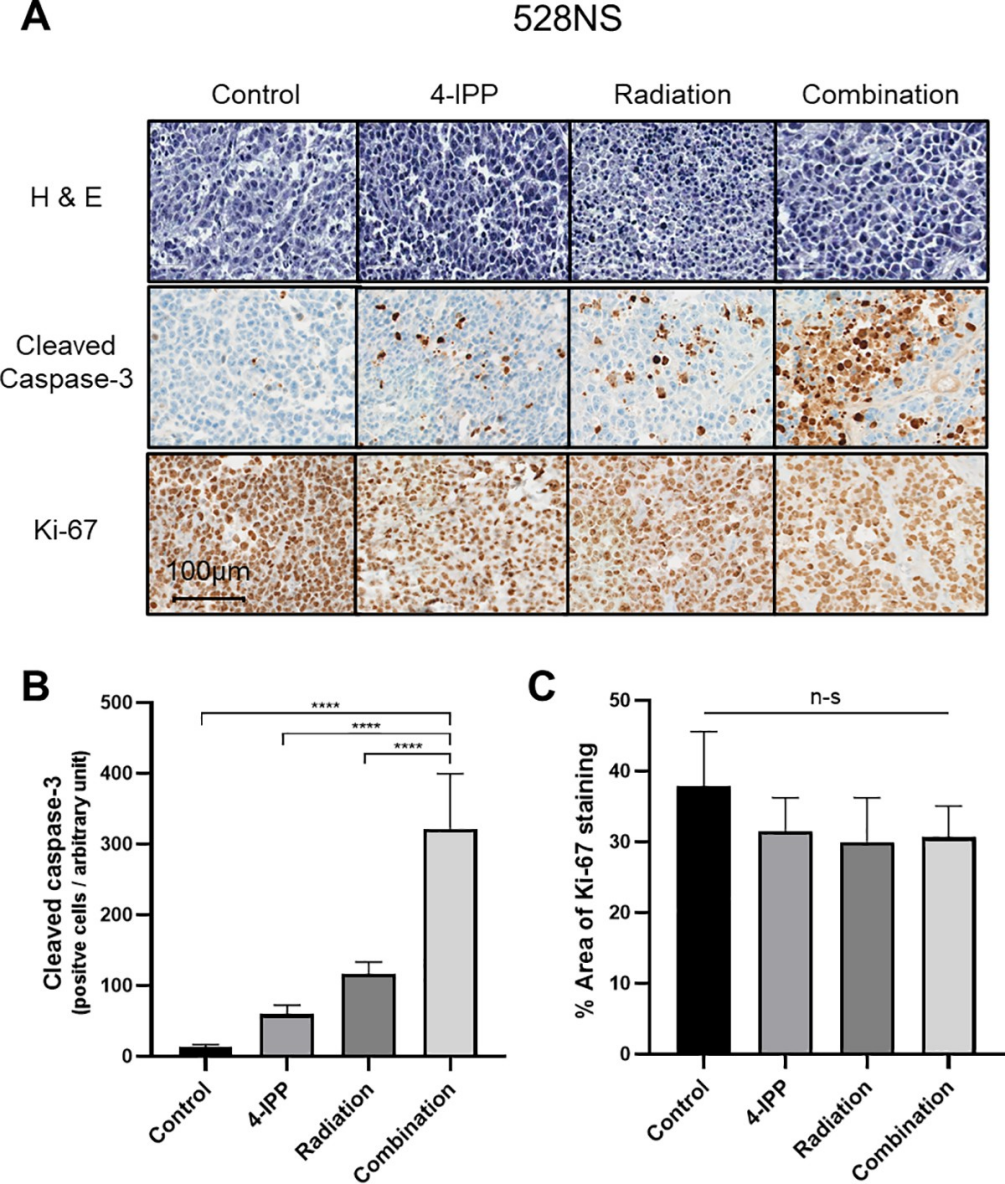
Cleaved caspase-3 and Ki-67 immunohistochemical stain on ex vivo tissue explants. (A) Representative images for cleaved caspase-3 and Ki-67 are shown. Scale bar, 100 μm. (B) The summary of the quantitative analysis results for cleaved caspase-3 and (C) Ki-67 is shown. The data represents the mean value of the number of cleaved caspase-3-positive cells per arbitrary unit (n = 5) and the percentage of Ki-67-positive stained area for each selected tissue area (n = 5). P values: **** p < 0.0001.

To examine whether 4-IPP had an influence on apoptosis or proliferation rate, we evaluated the apoptosis and proliferation rates by cleaved caspase-3 and Ki-67 staining, respectively (Fig 4A). The combination of 4-IPP and radiation showed a significant increase in cleaved caspase-3 staining, an indicator of apoptosis, compared to the 4-IPP and radiation monotherapy groups as well as the control group (Fig 4B). Treatment with 4-IPP and radiation trended to a lower percentage of Ki-67-positive stained area; however, the changes in values were not significant (Fig 4C).

### Effects of 4-IPP on suppressing tumor growth in a subcutaneous xenograft model

**Fig 5:**
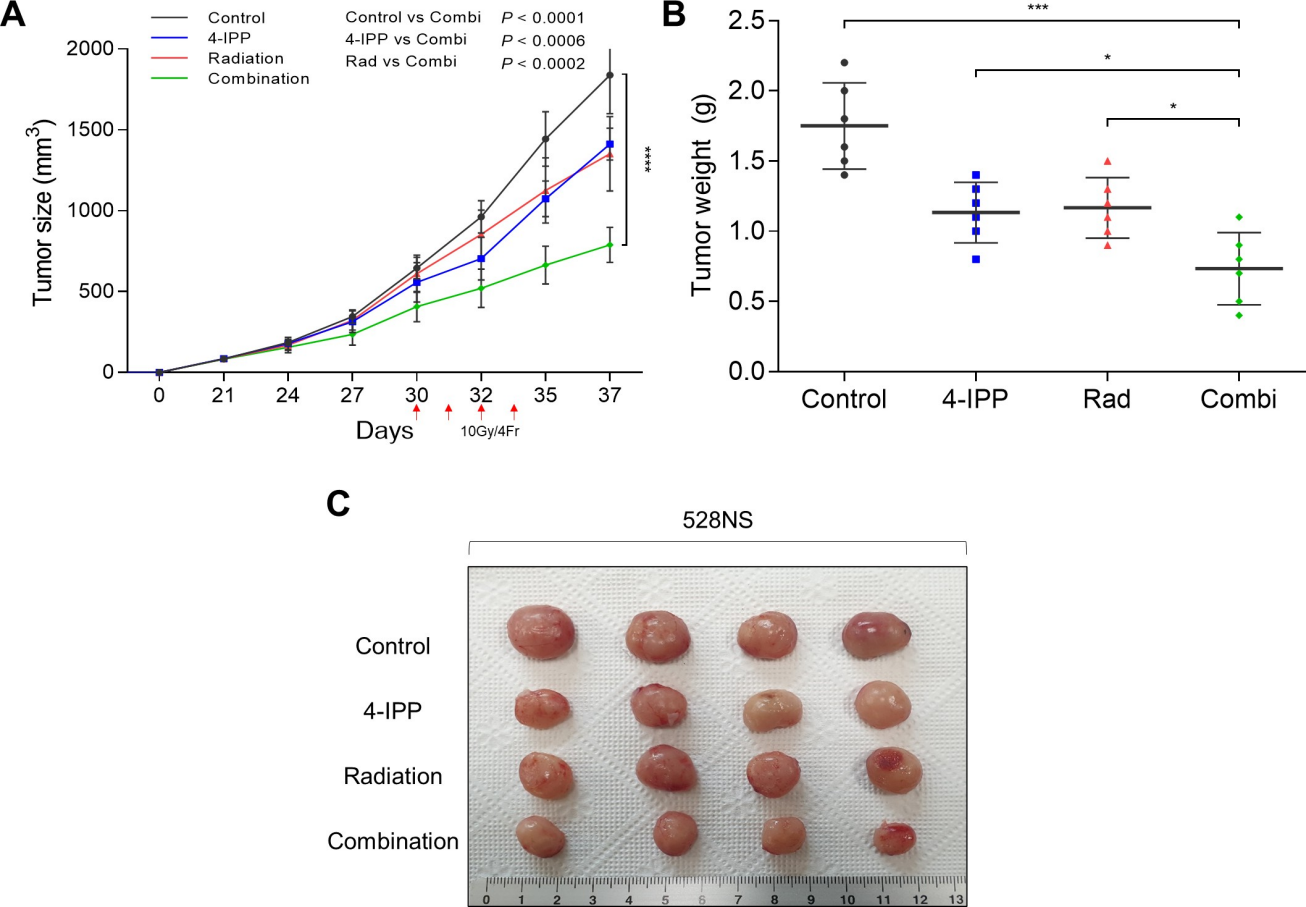
The effect of 4-IPP and radiation in the subcutaneous xenograft model. (A) Tumor size curves of different groups of mice during treatment. (B) Tumor weights and (C) photo of extracted tumors after treatment. P values: *p < 0.05, **p < 0.01, ***p < 0.001, **** p < 0.0001.

The effects of 54-IPP and radiation were evaluated in the subcutaneous xenograft model using 528NS cells. In contrast to the in vitro results, 4-IPP monotherapy was associated with moderate tumor growth suppression in the xenograft model, but when combined with radiation therapy, it was associated with a significant tumor growth delaying effect relative to each monotherapy regimen (Fig 5A–5C).

## Discussion

MIF plays a pleiotropic role in cancer promotion and recurrence [23]. Targeting MIF can improve the efficacy of radiation therapy and chemotherapy by inhibiting pro-tumorigenic properties and contributing to the restoration of immune sensitivity. Gupta et al. reported that mechanisms that mediate oxidative stress, such as ionizing radiation and other DNA-damaging substances, can induce the secretion of MIF into the extracellular environments of cells of kidney, breast, and lung cancer origins [5]. Also, Johler et al. reported that treatment with cytotoxic drugs increased the secretion of MIF in rhabdomyosarcoma cells, leading to immune escape [24]. Because increased MIF secretion promotes tumor cell survival, inflammation, and angiogenic complications, these reports highlight the benefits of adjuvant MIF inhibition to exhibit the full potential of anti-cancer therapy [5,24]. It has been reported that MIF inhibition can synergistically enhance the anti-cancer effects of common chemotherapy agents, such as temozolomide [25]. When the MIF inhibitor ibudilast was combined with temozolomide, significant synergism was observed, leading to cell cycle arrest and apoptosis, also resulting in prolonged overall survival in the in vivo model [25].

According to a study by Youn et al., MIF constructs a complex using ribosomal protein S3 in non-small cell lung cancer under conditions without irradiation [26]. However, when exposed to ionizing radiation, the complex dissociates, and the dissociated MIF activates NF-κB [26]. Besides, as mentioned above, MIF-TXNIP binding induces NF-κB activation and the expression of proliferative genes [17]. MIF induces NF-κB activity by offsetting the inhibitory effects of TXNIP on the NF-κB pathway via direct interaction with TXNIP [17].

Following radiotherapy, GSCs shift from the proneural to the mesenchymal subtype [27]. Mesenchymal trans-differentiation is driven by the activation of a limited set of master regulators, among which a key role is exploited by STAT3, C/EBP-β, and transcriptional coactivator with PDZ-binding motif (TAZ) [14,15]. These transcription factors regulate the self-renewal of mesenchymal GSCs and the expression of downstream signals [16]. NF-κB is the upstream regulator of these master regulators, and TGM2 is a key molecular mediator between NF-κB and the master transcription factors for necrosis-induced mesenchymal trans-differentiation [28]. In contrast with non-mesenchymal and early GBM samples, TGM2 expression is highly upregulated in recurrent mesenchymal patient samples and correlates with a poor prognosis [28]. After irradiation of proneural GSCs, TGM2 is upregulated via NF-κB signaling to enhance mesenchymal trans-differentiation and acquire radio-resistance [28]. Therefore, by suppressing MIF, we can expect effective downregulation of master regulators via the regulation of NF-κB and TGM2, and in our study, we confirmed that the increase in NF-κB and TGM2 after irradiation was suppressed by inhibiting MIF and DDT.

The MIF signaling cascade activates both the MAPK and PI3K pathways [18]. Both pathways are important intracellular signal transduction cascades concurrently activated in many cancers, regulating cell growth, proliferation, survival, mobility, and invasion [29,30]. Extracellular signal-regulated kinase (ERK) acts at the end of the MAPK pathway to mediate various cellular processes, and protein kinase B (AKT) is a primary mediator of the PI3K pathway [31]. Radiation can also simultaneously induce MAPK and PI3K signaling pathways, and these signaling pathways act conjointly to promote the development of radio-resistance in cancer cells [32]. We demonstrated that the expression of pERK and pAKT was maintained or increased in the radiation-alone treatment group compared with the control group, but it was maintained or decreased in the 4-IPP and combination treatment groups, respectively. This suggests that inhibition of MIF and DDT may contribute to radio-sensitization also via downregulation of the MAPK and PI3K signaling pathways.

MIF is also known to potently inhibit apoptosis. MIF inhibits p53-dependent gene expression and suppresses apoptosis associated with p53 activation [33]. The PI3K pathway also contributes to the suppression of apoptosis mediated by MIF [33]. As 4-IPP has previously been shown to induce apoptosis in thyroid carcinoma, we evaluated whether the inhibition of MIF and DDT by 4-IPP also results in enhanced apoptosis in glioma. Cleavage of caspase-3 is a key event in cell apoptosis and is a reliable indicator of apoptosis [34]. As shown from Fig 4A and 4B, we could observe the most pronounced expression of cleaved caspase-3 in the tumor tissue section of 4-IPP and radiation combination-treated mice. Our findings showed that the inhibition of the up-regulated MIF signaling cascade in glioma renders tumor cells susceptible to apoptosis, and which suggests that it may also promote radiation-induced apoptosis, thereby contributing to radio-sensitization.

In this study, radiation therapy alone moderately inhibited GSC proliferation, but the combination of radiation therapy with 4-IPP was associated with a high level of tumor suppression. The 528NS cells, a proneural subtype GSC, have shown mesenchymal trans-differentiation and significantly upregulated sphere-forming ability after irradiation compared to control 528NS cells in the previous study by Yin et al. [28]. In our result with 528NS cells, stemness factors, such as Olig2 and SOX2, were maintained in the radiation-alone group, but these factors were decreased in the groups in which 4-IPP was administered. TGM2 and NF-κB expression were higher in the radiation-alone group but decreased or did not change in the group in which 4-IPP was administered. Based on these results, we could hypothesize that 4-IPP has the potential to enhance the therapeutic effects of radiation in proneural GSCs by downregulating stemness and intracellular signaling pathways, thereby inducing apoptosis, and by decreasing the tendency toward mesenchymal trans-differentiation from MIF and DDT inhibition.

However, since this study was conducted using a subcutaneous xenograft model rather than an orthotopic xenograft model, there are clear limitations, and therefore, the results of this study need to be interpreted carefully. Previous literature by Mitchell et al. has briefly mentioned that 4-IPP is non-toxic, can be delivered orally, and crosses the blood-brain barrier (BBB) [35], but the authors have described this characteristic in a short sentence as an “unpublished observation” without presenting supporting data. Therefore, further studies on BBB permeability and the safety of 4-IPP should be conducted in the future. In addition, although it was not carried out in this study, further analysis seems to be needed to determine whether the effect of the 4-IPP and radiation combination is additive or synergistic.

In summary, our study showed that MIF and DDT are promising targets for overcoming radio-resistance in association with treatment and tumor recurrence. Current clinical strategies for GBM can be strengthened by adding molecular therapies targeting the MIF and DDT.

## Supporting information

S1 Checklist The ARRIVE guidelines 2.0: Author checklist. (PDF)

S1 Fig Evaluation of chemical interactions of 4-IPP and the MTT assay. The assays were performed as described in Materials and methods, and drugs were used at four different concentrations. Percentage variation indicates deviation from the blank group (0 μM). Results are presented as the mean value of nine experiments.
(TIFF)

S2 Fig MTT proliferation assay curves of 448T with various concentrations of 4-IPP. (TIFF)

S3 Fig The expression of proteins in the Western blot analysis. The effect of 4-IPP was evaluated by Western blot analysis. The expression levels of stemness factors were reduced in two kinds of GSCs (528NS and 448T) by 4-IPP in a dose-dependent manner.
(TIFF)

S1 Raw images (PDF)

## References

[1] Wen PY, Kesari S. Malignant gliomas in adults. N Engl J Med. 2008; 359:492–507. doi: 10.1056/NEJMra0708126 . 18669428

[2] Anjum K, Shagufta BI, Abbas SQ, Patel S, Khan I, Shah SAA, et al. Current status and future therapeutic perspectives of glioblastoma multiforme (GBM) therapy: A review. Biomed Pharmacother. 2017; 92:681–9. doi: 10.1016/j.biopha.2017.05.125 . 28582760

[3] Multhoff G, Radons J. Radiation, inflammation, and immune responses in cancer. Front Oncol. 2012; 2:58. doi: 10.3389/fonc.2012.00058. 22675673 PMC3366472

[4] Gupta K, Burns TC. Radiation-Induced Alterations in the Recurrent Glioblastoma Microenvironment: Therapeutic Implications. Front Oncol. 2018; 8:503. doi: 10.3389/fonc.2018.00503. 30467536 PMC6236021

[5] Gupta Y, Pasupuleti V, Du W, Welford SM. Macrophage Migration Inhibitory Factor Secretion Is Induced by Ionizing Radiation and Oxidative Stress in Cancer Cells. PLoS One. 2016; 11:e0146482. doi: 10.1371/journal.pone.0146482. 26741693 PMC4704778

[6] Mittelbronn M, Platten M, Zeiner P, Dombrowski Y, Frank B, Zachskorn C, et al. Macrophage migration inhibitory factor (MIF) expression in human malignant gliomas contributes to immune escape and tumour progression. Acta Neuropathol. 2011; 122:353–65. doi: 10.1007/s00401-011-0858-3 . 21773885

[7] Wang XB, Tian XY, Li Y, Li B, Li Z. Elevated expression of macrophage migration inhibitory factor correlates with tumor recurrence and poor prognosis of patients with gliomas. J Neurooncol. 2012; 106:43–51. doi: 10.1007/s11060-011-0640-3 . 21725855

[8] Goldman MJ, Craft B, Hastie M, Repecka K, McDade F, Kamath A, et al. Visualizing and interpreting cancer genomics data via the Xena platform. Nat Biotechnol. 2020; 38:675–8. doi: 10.1038/s41587-020-0546-8 . 32444850 PMC7386072

[9] Osuka S, Van Meir EG. Overcoming therapeutic resistance in glioblastoma: the way forward. J Clin Invest. 2017; 127:415–26. doi: 10.1172/JCI89587 . 28145904 PMC5272196

[10] Sakamoto D, Takagi T, Fujita M, Omura S, Yoshida Y, Iida T, et al. Basic Gene Expression Characteristics of Glioma Stem Cells and Human Glioblastoma. Anticancer Res. 2019; 39:597–607. doi: 10.21873/anticanres.13153 . 30711935

[11] Fukaya R, Ohta S, Yaguchi T, Matsuzaki Y, Sugihara E, Okano H, et al. MIF Maintains the Tumorigenic Capacity of Brain Tumor-Initiating Cells by Directly Inhibiting p53. Cancer Res. 2016; 76:2813–23. doi: 10.1158/0008-5472.CAN-15-1011 . 26980763

[12] Wang Z, Xue Y, Wang P, Zhu J, Ma J. MiR-608 inhibits the migration and invasion of glioma stem cells by targeting macrophage migration inhibitory factor. Oncol Rep. 2016; 35:2733–42. doi: 10.3892/or.2016.4652 . 26935642

[13] Otvos B, Silver DJ, Mulkearns-Hubert EE, Alvarado AG, Turaga SM, Sorensen MD, et al. Cancer Stem Cell-Secreted Macrophage Migration Inhibitory Factor Stimulates Myeloid Derived Suppressor Cell Function and Facilitates Glioblastoma Immune Evasion. Stem Cells. 2016; 34:2026–39. doi: 10.1002/stem.2393 . 27145382 PMC5820763

[14] Bhat KPL, Balasubramaniyan V, Vaillant B, Ezhilarasan R, Hummelink K, Hollingsworth F, et al. Mesenchymal differentiation mediated by NF-kappaB promotes radiation resistance in glioblastoma. Cancer Cell. 2013; 24:331–46. doi: 10.1016/j.ccr.2013.08.001 . 23993863 PMC3817560

[15] Fedele M, Cerchia L, Pegoraro S, Sgarra R, Manfioletti G. Proneural-Mesenchymal Transition: Phenotypic Plasticity to Acquire Multitherapy Resistance in Glioblastoma. Int J Mol Sci. 2019; 20 doi: 10.3390/ijms20112746. 31167470 PMC6600373

[16] Zanotto-Filho A, Goncalves RM, Klafke K, de Souza PO, Dillenburg FC, Carro L, et al. Inflammatory landscape of human brain tumors reveals an NFkappaB dependent cytokine pathway associated with mesenchymal glioblastoma. Cancer Lett. 2017; 390:176–87. doi: 10.1016/j.canlet.2016.12.015 . 28007636

[17] Kim MJ, Kim WS, Kim DO, Byun JE, Huy H, Lee SY, et al. Macrophage migration inhibitory factor interacts with thioredoxin-interacting protein and induces NF-kappaB activity. Cell Signal. 2017; 34:110–20. doi: 10.1016/j.cellsig.2017.03.007 . 28323005

[18] Trivedi-Parmar V, Jorgensen WL. Advances and Insights for Small Molecule Inhibition of Macrophage Migration Inhibitory Factor. J Med Chem. 2018; 61:8104–19. doi: 10.1021/acs.jmedchem.8b00589 . 29812929 PMC6311451

[19] Merk M, Zierow S, Leng L, Das R, Du X, Schulte W, et al. doi: 10.1073/pnas.1102941108. Proc Natl Acad Sci U S A. 2011; 108:E577–85. 10.1073/pnas.1102941108. 21817065 PMC3161582

[20] Pasupuleti V, Du W, Gupta Y, Yeh IJ, Montano M, Magi-Galuzzi C, et al. Dysregulated D-dopachrome tautomerase, a hypoxia-inducible factor-dependent gene, cooperates with macrophage migration inhibitory factor in renal tumorigenesis. J Biol Chem. 2014; 289:3713–23. doi: 10.1074/jbc.M113.500694 . 24356968 PMC3916569

[21] Rajasekaran D, Zierow S, Syed M, Bucala R, Bhandari V, Lolis EJ. Targeting distinct tautomerase sites of D-DT and MIF with a single molecule for inhibition of neutrophil lung recruitment. Faseb j. 2014; 28:4961–71. doi: 10.1096/fj.14-256636 . 25016026 PMC4200328

[22] Ulukaya E, Colakogullari M, Wood EJ. Interference by anti-cancer chemotherapeutic agents in the MTT-tumor chemosensitivity assay. Chemotherapy. 2004; 50:43–50. doi: 10.1159/000077285 . 15084806

[23] Mitchell RA, Yaddanapudi K. Stromal-dependent tumor promotion by MIF family members. Cell Signal. 2014; 26:2969–78. doi: 10.1016/j.cellsig.2014.09.012 . 25277536 PMC4293307

[24] Johler SM, Fuchs J, Seitz G, Armeanu-Ebinger S. Macrophage migration inhibitory factor (MIF) is induced by cytotoxic drugs and is involved in immune escape and migration in childhood rhabdomyosarcoma. Cancer Immunol Immunother. 2016; 65:1465–76. doi: 10.1007/s00262-016-1896-4 . 27629595 PMC11029580

[25] Ha W, Sevim-Nalkiran H, Zaman AM, Matsuda K, Khasraw M, Nowak AK, et al. Ibudilast sensitizes glioblastoma to temozolomide by targeting Macrophage Migration Inhibitory Factor (MIF). Sci Rep. 2019; 9:2905. doi: 10.1038/s41598-019-39427-4. 30814573 PMC6393433

[26] Youn H, Son B, Kim W, Jun SY, Lee JS, Lee JM, et al. Dissociation of MIF-rpS3 complex and sequential NF-kappaB activation is involved in IR-induced metastatic conversion of NSCLC. J Cell Biochem. 2015; 116:2504–16. doi: 10.1002/jcb.25195 . 25900216

[27] Kim SH, Ezhilarasan R, Phillips E, Gallego-Perez D, Sparks A, Taylor D, et al. Serine/Threonine Kinase MLK4 Determines Mesenchymal Identity in Glioma Stem Cells in an NF-kappaB-dependent Manner. Cancer Cell. 2016; 29:201–13. doi: 10.1016/j.ccell.2016.01.005 . 26859459 PMC4837946

[28] Yin J, Oh YT, Kim JY, Kim SS, Choi E, Kim TH, et al. Transglutaminase 2 Inhibition Reverses Mesenchymal Transdifferentiation of Glioma Stem Cells by Regulating C/EBPbeta Signaling. Cancer Res. 2017; 77:4973–84. doi: 10.1158/0008-5472.CAN-17-0388 . 28754668

[29] McCubrey JA, Steelman LS, Chappell WH, Abrams SL, Wong EW, Chang F, et al. Roles of the Raf/MEK/ERK pathway in cell growth, malignant transformation and drug resistance. Biochim Biophys Acta. 2007; 1773:1263–84. doi: 10.1016/j.bbamcr.2006.10.001 . 17126425 PMC2696318

[30] Manning BD, Toker A. AKT/PKB signaling: navigating the network. Cell. 2017; 169:381–405. doi: 10.1016/j.cell.2017.04.001 . 28431241 PMC5546324

[31] Cao Z, Liao Q, Su M, Huang K, Jin J, Cao D. AKT and ERK dual inhibitors: the way forward? Cancer Lett. 2019; 459:30–40. doi: 10.1016/j.canlet.2019.05.025 . 31128213

[32] Hein AL, Ouellette MM, Yan Y. Radiation-induced signaling pathways that promote cancer cell survival. Int J Oncol. 2014; 45:1813–9. doi: 10.3892/ijo.2014.2614 . 25174607 PMC4203326

[33] Lue H, Thiele M, Franz J, Dahl E, Speckgens S, Leng L, et al. Macrophage migration inhibitory factor (MIF) promotes cell survival by activation of the Akt pathway and role for CSN5/JAB1 in the control of autocrine MIF activity. Oncogene. 2007; 26:5046–59. doi: 10.1038/sj.onc.1210318 . 17310986

[34] Porter AG, Jänicke RU. Emerging roles of caspase-3 in apoptosis. Cell Death Differ. 1999; 6:99–104. doi: 10.1038/sj.cdd.4400476 . 10200555

[35] Mitchell RA. Hypoxic Adaptation Facilitated by MIF. In: Bucala R, editor. The MIF Handbook. Singapore: World Scientific; 2012. pp. 161–81.

