# Supplementary figures and images for: Macrophage migration inhibitory factor (MIF) inhibitor 4-IPP downregulates stemness phenotype and mesenchymal trans-differentiation after irradiation in glioblastoma multiforme
Source: PLoS One. 2021 Sep 13;16(9):e0257375. doi: 10.1371/journal.pone.0257375 (PMC8437287; doi:10.1371/journal.pone.0257375)

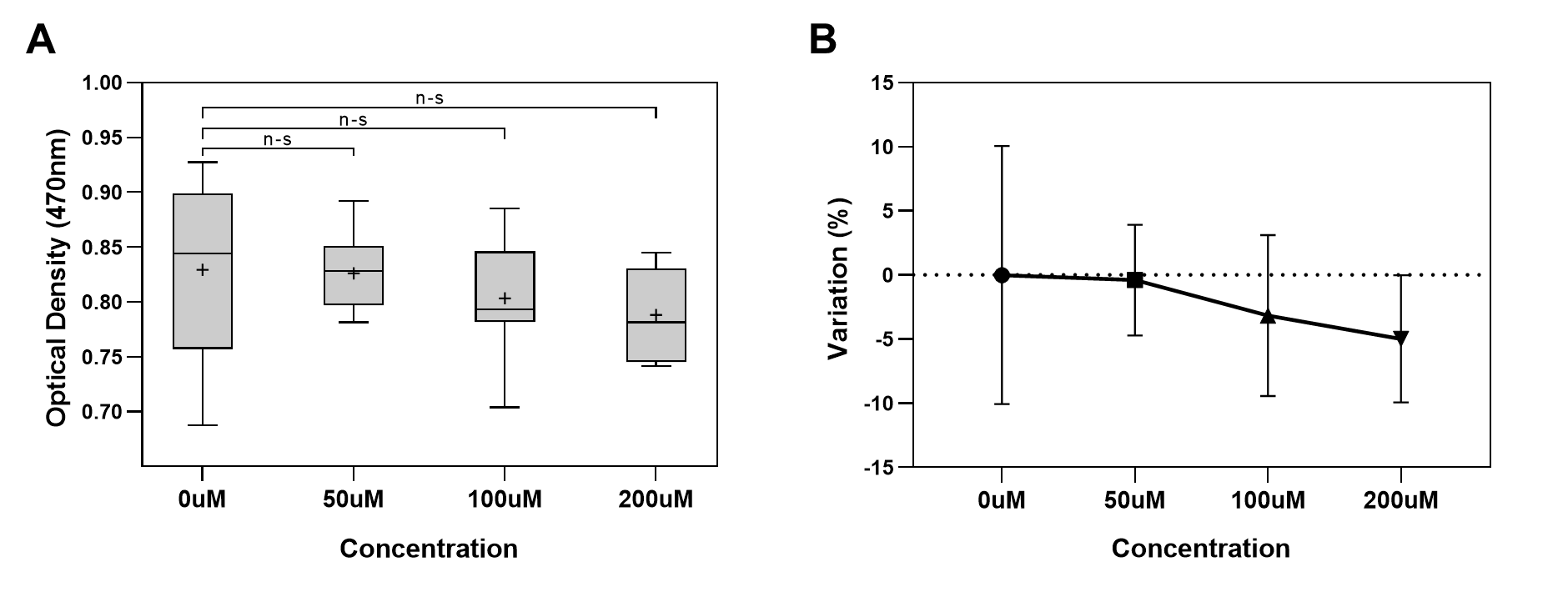

Supplement: S1 Fig — The assays were performed as described in Materials and methods, and drugs were used at four different concentrations. Percentage variation indicates deviation from the blank group (0 μM). Results are presented as the mean value of nine experiments. (TIFF) [file pone.0257375.s002.tiff]

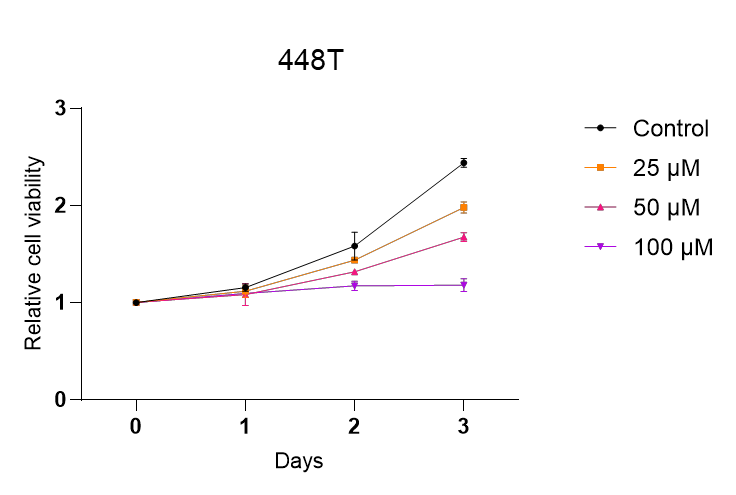

Supplement: S2 Fig — (TIFF) [file pone.0257375.s003.tiff]

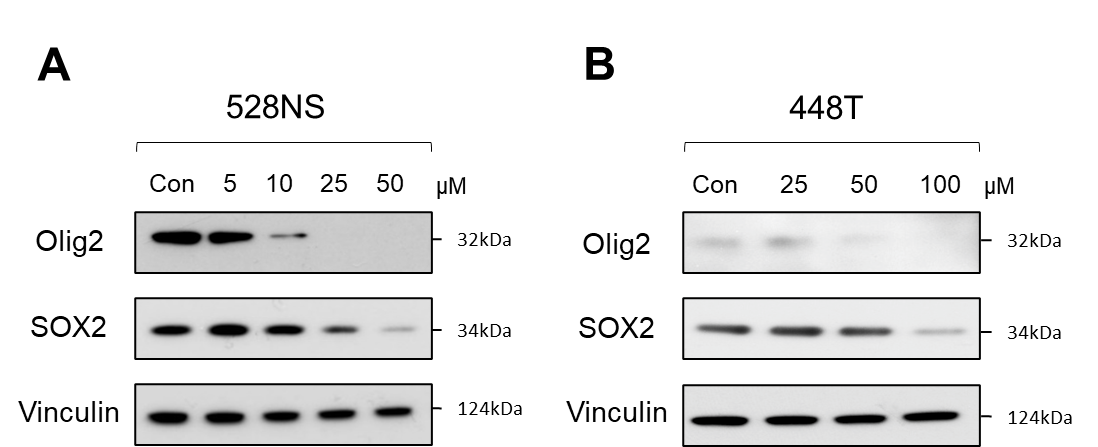

Supplement: S3 Fig — The effect of 4-IPP was evaluated by Western blot analysis. The expression levels of stemness factors were reduced in two kinds of GSCs (528NS and 448T) by 4-IPP in a dose-dependent manner. (TIFF) [file pone.0257375.s004.tiff]
