# Supplementary material for: Macrophage migration inhibitory factor (MIF) inhibitor 4-IPP downregulates stemness phenotype and mesenchymal trans-differentiation after irradiation in glioblastoma multiforme
Source: PLoS One. 2021 Sep 13;16(9):e0257375. doi: 10.1371/journal.pone.0257375 (PMC8437287; doi:10.1371/journal.pone.0257375)
Supplement: S1 Raw images — (PDF) [file pone.0257375.s005.pdf]

# Western blot scans

**Fig 2.**

528NS
Control, 4-IPP, Radiation, Combination treatment

Olig2, MIF, Vinculin

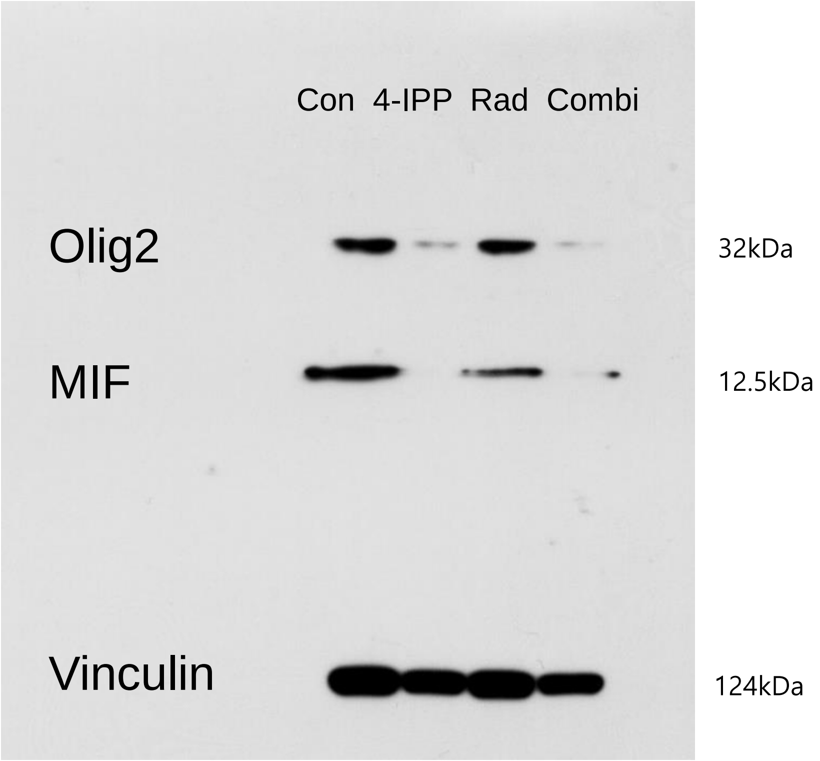


High exposure

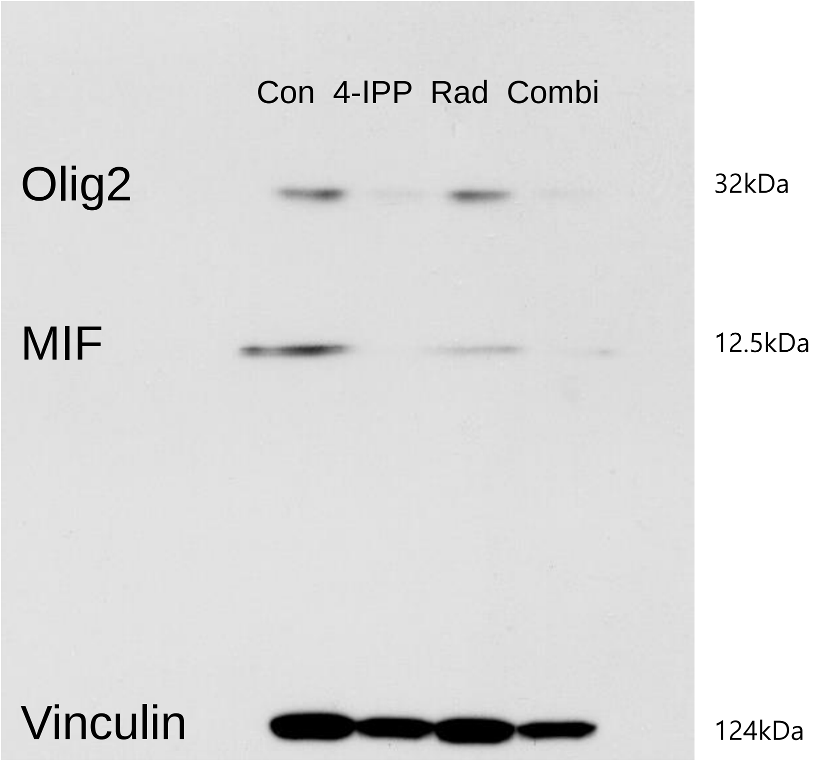


Low exposure

**Fig 2.**

528NS
Control, 4-IPP, Radiation, Combination treatment

pAKT, pERK

pAKT
pERK

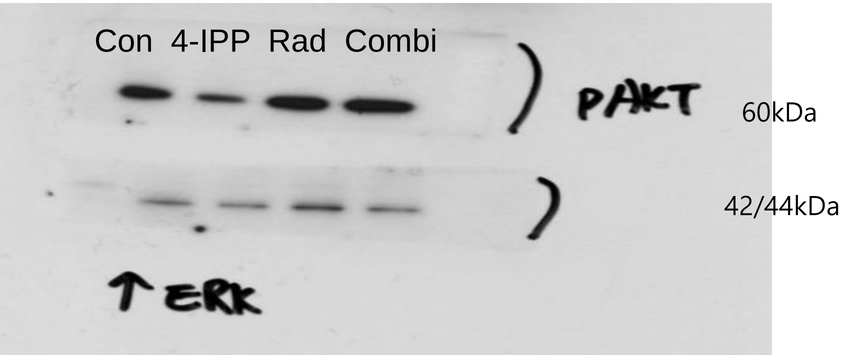


60kDa
42/44kDa

High exposure

pAKT
pERK

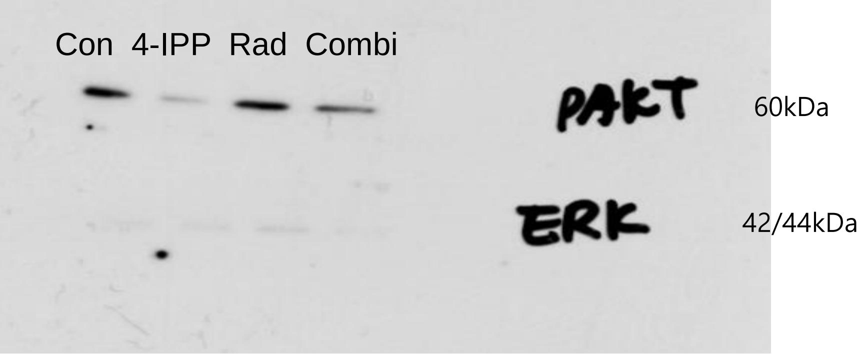


60kDa
42/44kDa

Low exposure

**Fig 2.**

528NS
Control, 4-IPP, Radiation, Combination treatment

SOX2, Vinculin

SOX2

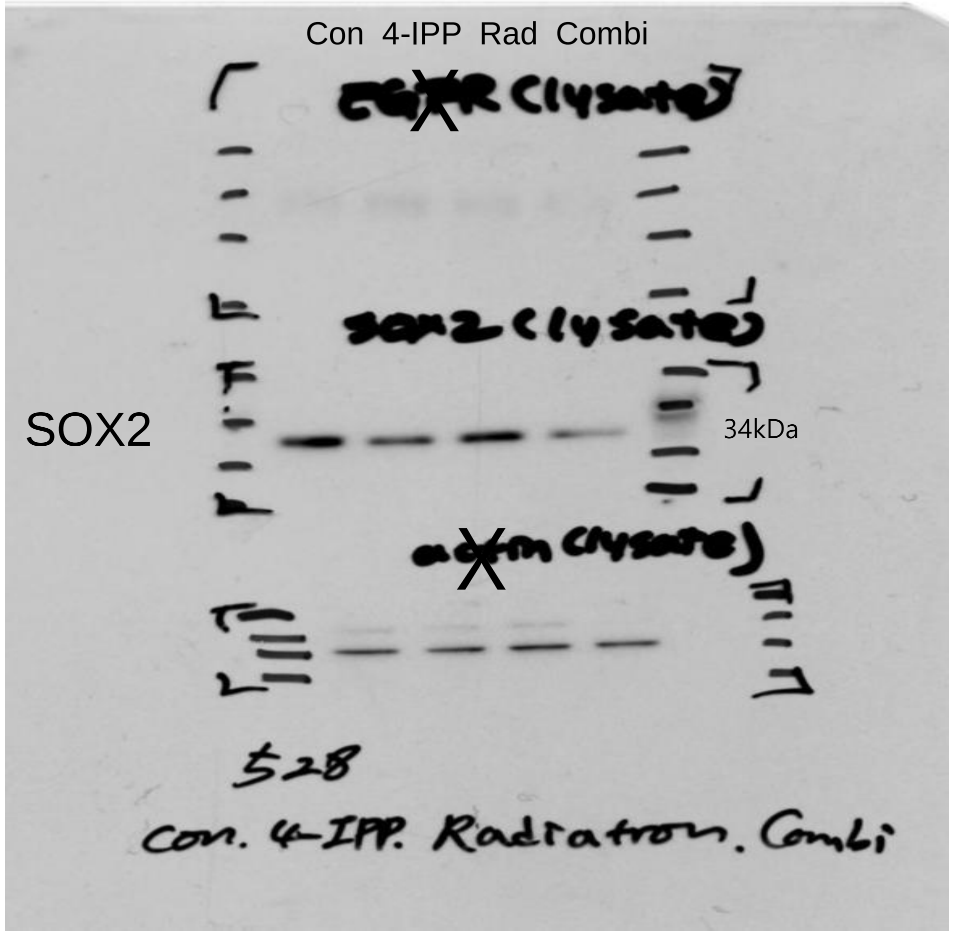


Vinculin

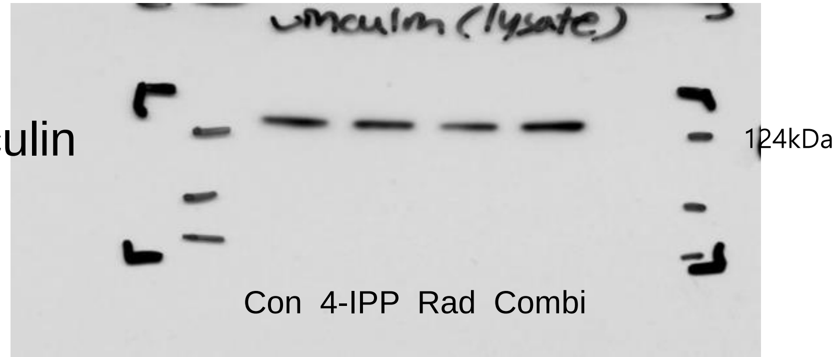

**Fig 2.**

528NS
Control, 4-IPP, Radiation, Combination treatment

TGM2, NF-κB, C/EBP-β, β-actin

NF-κB

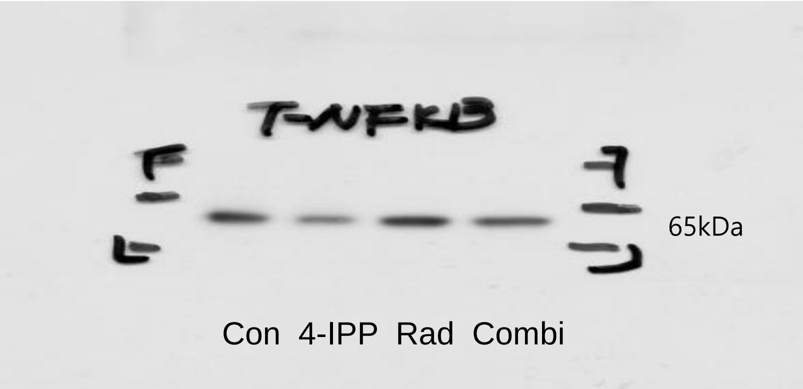


65kDa

TGM2

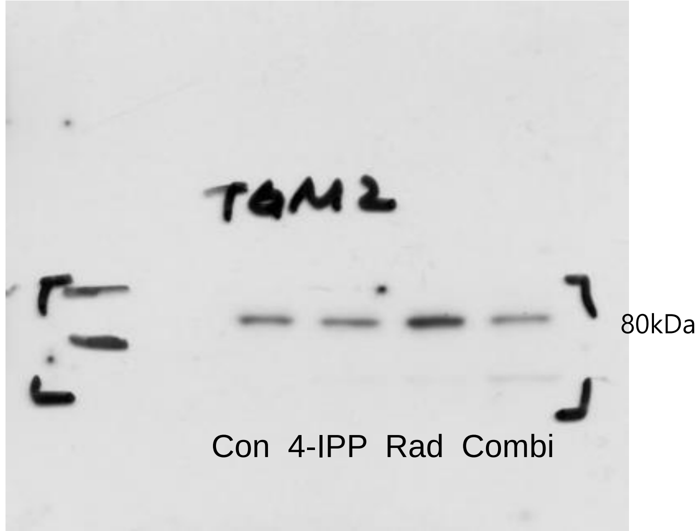


80kDa

C/EBP-β

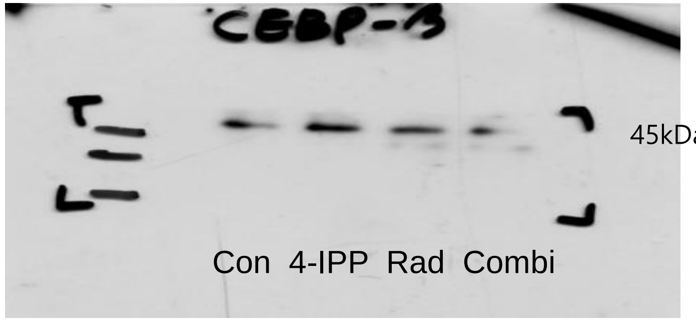


45kDa

β-actin

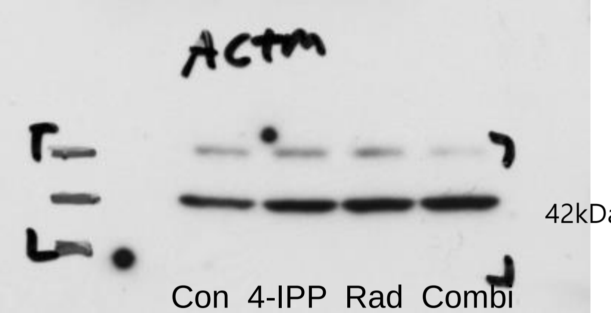


42kDa

**S3 Fig.**

528NS
Control, 5μM, 10μM, 25μM, 50μM treatment (4-IPP)

Olig2, SOX2, Vinculin

Olig2

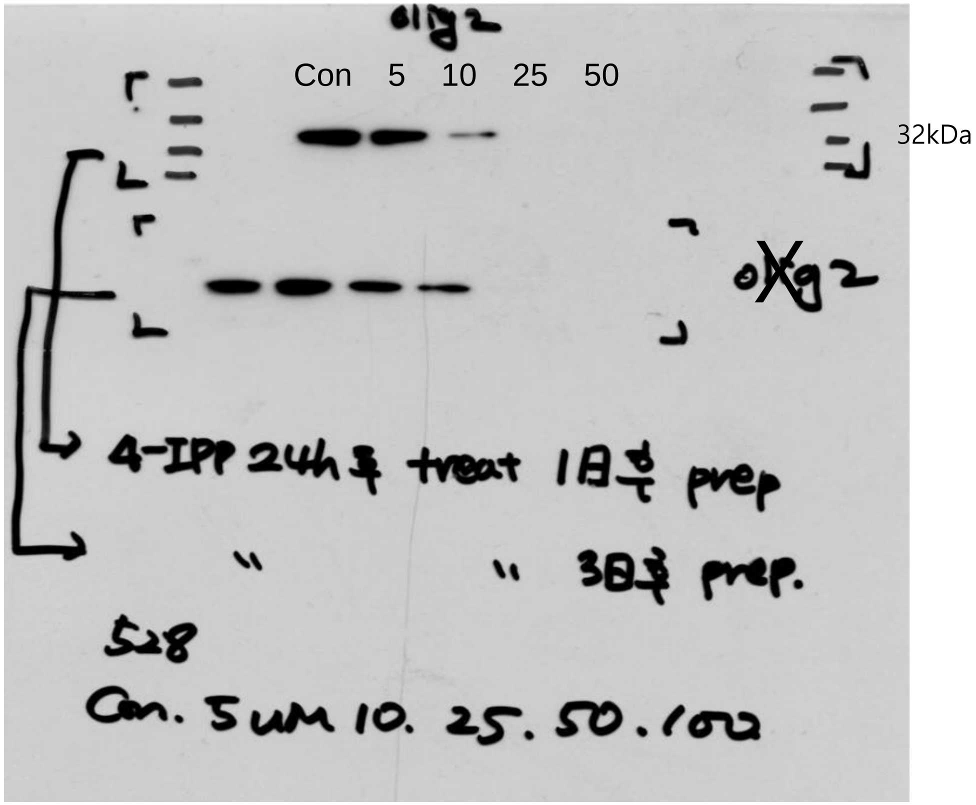


32kDa

SOX2

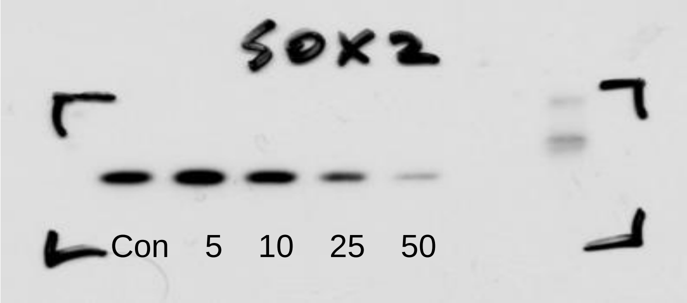


34kDa

Vinculin

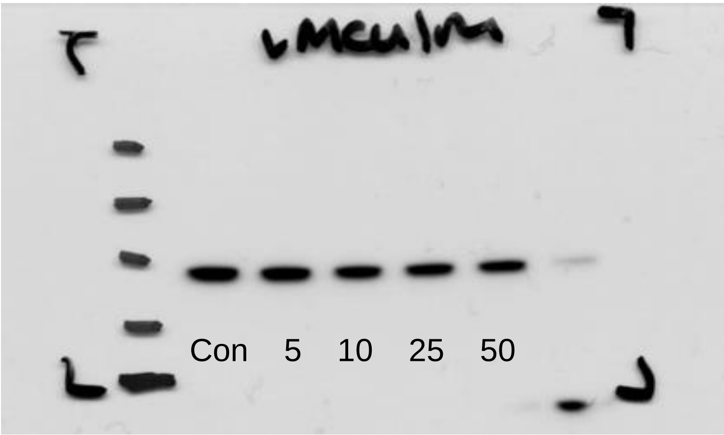


124kDa

**S3 Fig.**

448T
Control, 25μM, 50μM, 100μM treatment (4-IPP)

Olig2, SOX2, Vinculin

SOX2

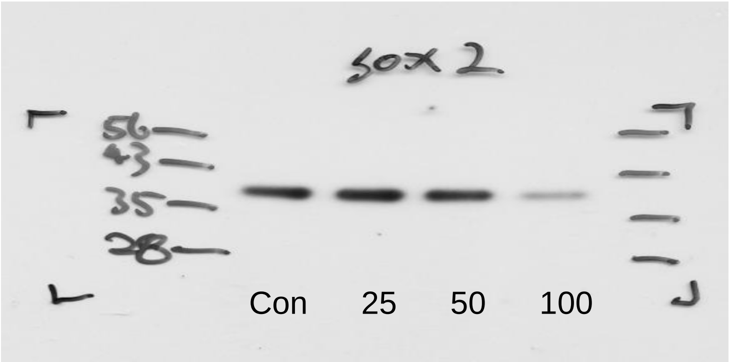


34kDa

Olig2

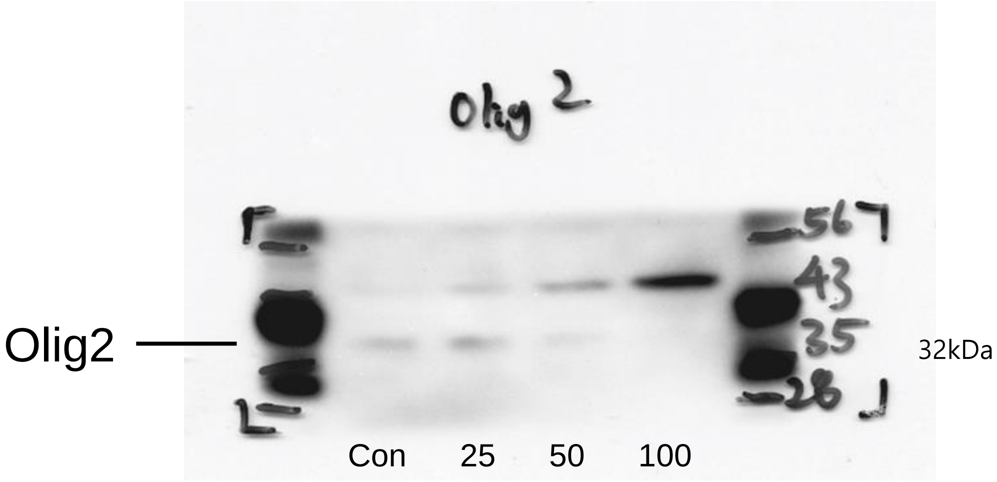


32kDa

Vinculin

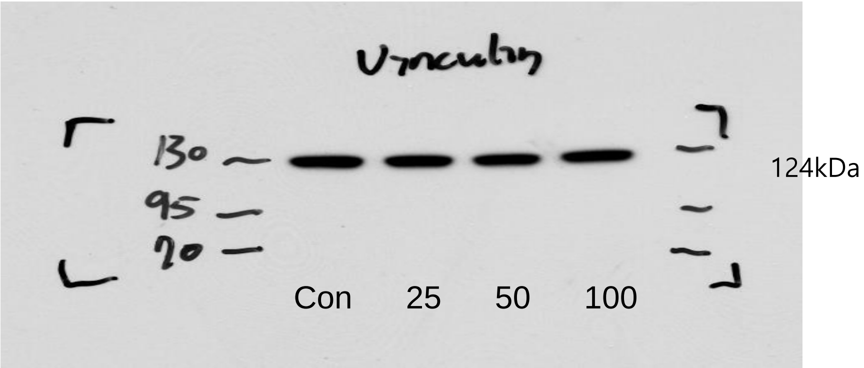


124kDa
